# A Novel Potentiometric Sensor for Determination of Neurotoxin **β**-*N*-Oxalyl-L-**α**, **β**-Diaminopropionic Acid

**DOI:** 10.1155/2014/251653

**Published:** 2014-05-20

**Authors:** Omer Isildak, Furkan Saymaz, Ahmet Karadag, Nesrin Okumus Korkmaz, Azade Attar

**Affiliations:** ^1^Department of Chemistry, Faculty of Science and Arts, Gaziosmanpasa University, 60240 Tokat, Turkey; ^2^Department of Bioengineering, Faculty of Chemical and Metallurgical Engineering, Yıldız Technical University, Esenler, 34210 Istanbul, Turkey

## Abstract

A novel potentiometric sensor based on ionophore (Cd(NH_2_CH_2_CH_2_OCH_2_CH_2_OCH_2_CH_2_NH_2_)Ag_3_(CN)_5_) for the determination of **β**-*N*-oxalyl-l-**α**, **β**-diaminopropionic acid (ODAP) is developed. The ODAP-selective membrane sensor demonstrates high sensitivity and short response time. The detection limit of the ODAP-selective membrane sensor is about 2 × 10^−6^ mol L^*-1*^ and the response time is shorter than 6 s. The linear dynamic range of the ODAP-selective membrane sensor is between ODAP concentrations of 1.0 × 10^−2^ and 1 × 10^−6^ mol L^*-1*^. The ODAP-selective membrane sensor exhibits good operational stability for at least one week in dry conditions at 4–6°C. It has a reproducible and stable response during continuous work for at least 10 h with a relative standard deviation of 0.28% (n = 18).

## 1. Introduction

Neurolathyrism is caused by a neuroexcitatory nonprotein amino acid, *β*-*N*-oxalyl-l-*α*, *β*-diaminopropionic acid (ODAP) [1–3]. This disease is characterized by a spastic paraplegia whose sporadic outbreaks have occurred at times due to the excessive consumption of the pulse* Lathyrus sativus* (grass pea, khesari dal, guaya, and chickling pea) especially under famine conditions in certain parts of Bangladesh, Ethiopia, Spain, Russia, and India [4–6]. The major toxic component of the pulse is ODAP, a neurotoxin amino acid [3, 4], which can potentially act as an agonist at certain glutamate receptors and [7, 8] inhibit specifically tyrosine aminotransferase [9] and chelates metals like zinc [10] that is also present in the longevity promoting Ginseng root [11]. However, the cultivation of the* Lathyrus sativus, *which contains high amount of ODAP, has been continuing in several parts of the world, and large populations still consume it in their daily diet [4]. Even under severe drought conditions when* Lathyrus sativus* has been consumed as the sole dietary source, the disease affects only less than 5% of the individuals and more than 95% always escape from any ill effects of the pulse [4, 12].

Recently, a few HPLC methods for detection of ODAP have been developed [1, 13–16]. Intensive research for developing low- or zero-neurotoxin ODAP of* Lathyrus sativus* seeds is continuing in several institutes focusing on agricultural reserves [13]. The most obvious option to achieve this goal entails plant breeding and postharvest analysing. This involves processing of a large number of samples, requiring a fast and selective method for monitoring ODAP.

9-Fluorenylmethyl chloroformate (FMOC) derivatization can be used for the detection and quantitative estimation of amounts of ODAP [17]. Euerby et al. separated the D and L isomers of ODAP with* o*-phthaldialdehyde chiral thiols by reversed-phase chromatography, using fluorescence detection, but the procedures are time consuming [18]. Khan et al. used precolumn derivatization with phenyl isothiocyanate (PITC) [19, 20]. The most common method utilizes the reaction of* o*-phthalaldehyde (OPA) with 2,3-diaminopropionic acid (DAP) formed on hydrolysis of both *α*- and *β*-isomers of ODAP, and the derivative is detected at 420 nm [1, 17]. However, the method cannot differentiate *α*- and *β*-ODAP and both are detected.

**Figure 1:**
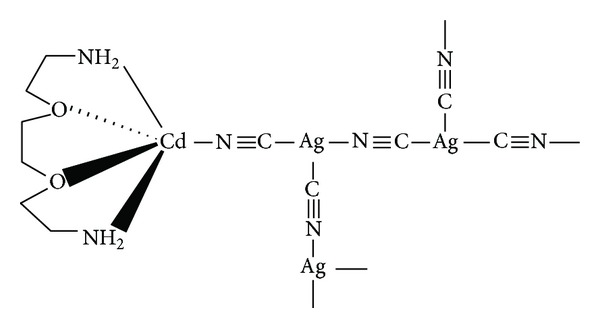
The structure of the ionophore.

To the best of our knowledge, as alternatives to the present methods for ODAP determination, no previous work has been reported on the potentiometric ODAP-selective membrane sensor to date. Therefore, the present study reports the development of a potentiometric ODAP-selective membrane sensor based on the ionophore (cadmium(2-[2-(2-aminoethoxy)ethoxy]ethan-1-amine) pentacyano trisilver) which is shown in Figure 1. The main analytical characteristics of the sensor, such as pH behavior and the amount of the ionophore, were investigated with respect to the influence on sensitivity, selectivity, limit of detection, dynamic range, response time, operation, and storage stability.

## 2. Material and Methods

### 2.1. Materials

All of the reagents used were analytical reagent grade and double distilled deionized water was used throughout. The *α*-ODAP and *β*-ODAP mixture was purified from* Lathyrus sativus *extracts as described previously [21] and used throughout the study as mixture. High molecular mass poly(vinyl chloride) (PVC), o-nitrophenyloctylether (o-NPOE), potassium tetrakis (p-chlorophenyl) borate (KTpClPB), tetrahydrofuran (THF), and graphite were obtained from Fluka. Epoxy resin (Macroplast Su 2227) and hardener (Desmodur RFE) were obtained from Henkel and Bayer, respectively. Di-cyclo-hexyl 18-crown-6-NaI salt was synthesized according to Pedersen's method [22]. ODAP-selective ionophore [Cd(NH_2_CH_2_CH_2_OCH_2_CH_2_OCH_2_CH_2_NH_2_)Ag_3_(CN)_5_] was synthesized in the laboratory [23]. Deionized water was obtained by means of a DI 800 Model deionized water system.

A stock ODAP solution (1.0 × 10^−2 ^mol L^−1^) was prepared in distilled deionized water. The diluted solutions (1.0 × 10^−2^ to 1.0 × 10^−6 ^mol L^−1^) of ODAP were prepared by an appropriate dilution of the stock solution. All of the other reagents used were of analytical reagent grade. Twice-distilled deionized water was used throughout the study.

### 2.2. Synthesis of Ionophore

1 mmol of AgNO_3_ and 2 mmol of KCN are dissolved in distilled water-alcohol mixture resulting formation of dicyanosilver anion. 1 mmol of CdSO_4_·8/3H_2_O is added to this solution result a blurry solution and this solution was stirred and heated on a magnetic stirrer. And 2-[2-(2-aminoethoxy)ethoxy]ethan-1-amine alternatively named 2,2′-(ethylenedioxy)bis (ethylamine), H_2_N(CH_2_)_2_O(CH_2_)_2_O(CH_2_)_2_NH_2_ is added to this solution. Ethanol is added to this mixture and stirred at 60°C for 1 hour. The resulting mixture is filtered and the remaining solution is left at room temperature for crystal formation.

### 2.3. Apparatus

Potentiometric measurements were conducted at room temperature (20 ± 1°C) by using a multichannel potentiometric system (provided by Isedo medical instruments, Turkey) controlled by a computer and a homemade C-sharp programme was used. The potential values as steady-state responses of the ODAP-selective membrane sensor were performed for different concentrations of standard solutions of ODAP, respectively. Throughout the measurements, a microsized solid silver/silver chloride electrode (provided by Isedo medical instruments, Turkey) was used as reference electrode with the ODAP-selective membrane sensor. To investigate the potentiometric characteristics of the prepared ODAP-selective membrane sensor, measurements of the potential differences between two points which are the cause of electrical currents were taken in the following cell assembly: microsized solid silver/silver chloride reference electrode/test solution/ODAP-selective membrane sensor membrane/all solid-state contact material/Cu wire.

### 2.4. Design of All Solid-State Contact ODAP-Selective Membrane Sensor

The potentiometric all solid-state contact ODAP-selective membrane sensor was prepared as previously described in the literature [24]. The epoxy resin mixture used to bind the graphite in preparing the all solid-state contact of the sensor was made from epoxy and hardener in THF solvent in the proportions 1.0 : 0.5 w/w. The powdered graphite was mixed with the epoxy resin mixture in the proportions 1.0 : 1.0 w/w. After mixing, the solution was allowed to stand for 20–30 min in air. When the appropriate viscosity was attained, a shielded copper wire was dipped in the mixture several times to obtain solid-state contact uniformly coated and allowed to stand overnight in an oven at 40°C.

The ODAP-selective membrane solution which comprised ODAP ionophore (2.5%, w/w), di-cyclo-hexyl 18-crown-6-NaI (1.5%, w/w), o-NPOE (65.0%, w/w), KTpClPB (0.5%, w/w), and PVC (30.5%, w/w) was dissolved in 4 mL of THF. Di-cyclo-hexyl 18-crown-6-NaI salt was used within the membrane content to increase the ionic mobility of the membrane toward ODAP [25].

The solid-state contact was dipped into the ODAP-selective membrane solution at least three times and then the coated membrane was allowed to dry in the air for at least 3 hours. Finally, the prepared all solid-state contact ODAP-selective membrane sensor was soaked in a 0.01 M ODAP solution for at least 3 hours before use. The potentiometric performance characteristics of the all solid-state contact ODAP-selective membrane sensor were tested in steady-state conditions.

## 3. Results and Discussion

### 3.1. Potentiometric Performance of the All Solid-State Contact ODAP-Selective Membrane Sensor

As can be seen from results presented in Table 1, the all solid-state contact ODAP-selective membrane sensor based on (Cd(NH_2_CH_2_CH_2_OCH_2_CH_2_OCH_2_CH_2_NH_2_)Ag_3_(CN)_5_) (2.5%, w/w), di-cyclo-hexyl 18-crown-6-NaI (1.5%, w/w), together with other constituents, demonstrates very good selectivity towards ODAP over other ions tested. The potentiometric selectivity coefficients of all solid-state ODAP-selective membrane sensors were evaluated by the separate solutions method according to the IUPAC recommendation [26].

Potentiometric performance of the ODAP-selective membrane sensor was evaluated to optimize membrane composition. The potentiometric results obtained by evaluated ODAP-selective membrane compositions are summarized in Table 1. The performance of the potentiometric sensor, in terms of sensitivity and long-term stability, is strictly dependent on the amount of the ODAP-selective ionophore in membrane content. For the optimization of the amount of the ionophore in the membrane, different ionophore concentrations in the membrane solution were prepared. The highest sensitivity of potentiometric ODAP-sensor was observed when 2.5% (w/w) ionophore was loaded. The further increase in the amount of ionophore loading might lead to increase of the diffusion resistance for the ODAP to arrive to the electrode surface and then to the decrease in the sensor response. On the other hand, if the ionophore concentrations are lower than 1.0% (w/w), there is no enough ionophore involved in the reaction which leads only to a slight potential variation. As a result, the ionophore ratio of 2.5% (w/w) was used to obtain optimum membrane composition for further experiments.

**Figure 2:**
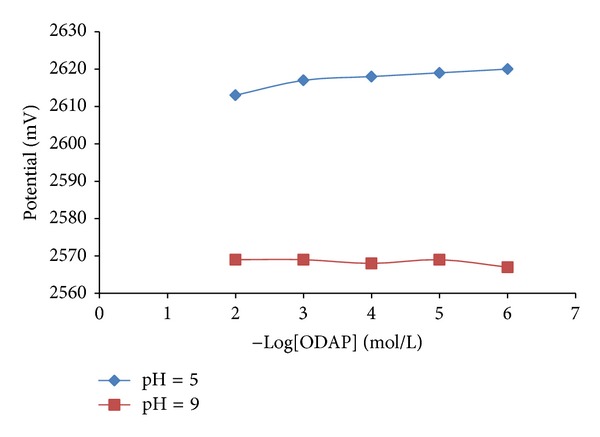
The dependence of the all solid-state contact ODAP-selective membrane sensor response in acidic and basic phosphate buffers.

Since ionophore activity is strongly affected by the pH of the solution, the effect of pH on the potentiometric ODAP-selective membrane sensor response was examined by using phosphate buffer systems (5 mM) at pH 5.00 and 9.00. At these pH solutions, the ODAP-selective membrane sensor exhibited almost no potential change in concentration range of 1.0 × 10^−2^ to 1.0 × 10^−6 ^mol L^−1^ of ODAP, respectively (Figure 2). From the obtained results we decided to use the potentiometric ODAP-selective membrane sensor at pH 7. Consequently neutral medium was prepared as much as possible as optimum working conditions for further experiments.

### 3.2. Analytical Characteristics of the All Solid-State Contact ODAP-Selective Membrane Sensor

**Figure 3:**
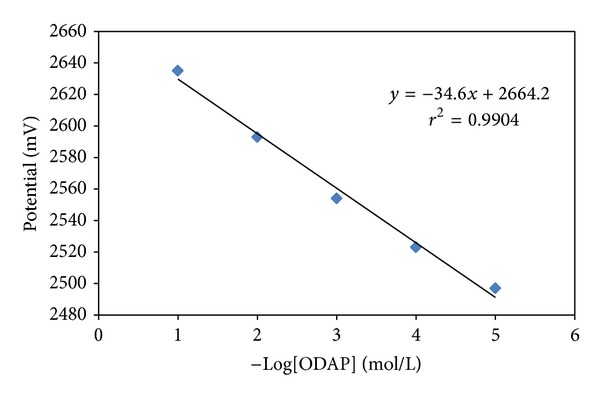
Calibration curve for the all solid-state contact ODAP-selective membrane sensor at pH: 7.

Using the optimum conditions determined in the above studies, calibration curve of the ODAP-selective membrane sensor was obtained over an ODAP concentration range of 1.0 × 10^−2^ to 1.0 × 10^−6 ^mol L^−1^. As shown in Figure 3, the ODAP-selective membrane sensor exhibited a linear response, and the graph of the linear response was defined by the equation of *E* = −34.6 Log *a*_ODAP_ + 2664.2 with a correlation coefficient *r* ^2^ = 0.9981. The limit of detection for ODAP-selective membrane sensor calculated was about 1.2 × 10^−6 ^mol L^−1^.

**Figure 4:**
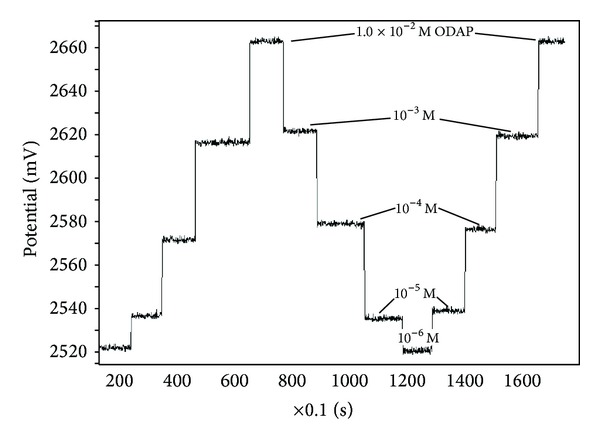
The response and reproducibility of the all solid-state contact ODAP-selective membrane sensor at pH: 7.

The response of the ODAP-selective membrane sensor was highly reproducible as shown in Figure 4. The novel ODAP-selective membrane sensor also exhibited good operational and storage stability. The potentiometric values obtained over an interval time of 15 minute showed that the ODAP-selective membrane sensor responses were reproducible and stable during continuous work at least for 10 h. Continuous and brakeless measurements could be done during this 10-hour period.

The relative standard deviation of ODAP-selective membrane sensor responses was approximately 0.28% (*n* = 18) for ODAP concentration of 1.0 × 10^−3 ^mol L^−1^. An investigation about long-term storage stability of ODAP-selective membrane sensor, kept in dry at 4–6°C, the calibration curve was recorded every day and the potentials were measured. The sensor demonstrated good storage stability for at least one week to standard ODAP concentration change. However, after one week there was a decrease of 20% from the initial potential. After 2 weeks, the decrease of the biosensor response was lower than 70% of its original response. These results indicate a less operational and storage stability of the ODAP-selective membrane sensor than those of potentiometric sensors in the studies [27–29]. The ODAP-selective membrane sensor reached a steady-state rapidly (Table 1), and its detection limit was 1.15 × 10^−6 ^mol L^−1^ which is better than those in the literature [28, 30], as the lowest limit in the linear range was 8.0 × 10^−6 ^mol L^−1^. The ODAP-selective membrane sensor presented in this work also exhibited a short response time less than 6 s better than those in the studies [28, 31], wider working concentration ranges 1.0 × 10^−1^ to 8.0 × 10^−6 ^mol L^−1^, and unfortunately short life span when compared to those of the sensors in other studies [29, 32, 33].

## 4. Conclusion

A novel all solid-state contact potentiometric ODAP-selective membrane sensor for the determination of ODAP is developed. All solid-state ODAP-selective membrane sensor presented here is cheap and easy to fabricate. Moreover, the sensor has a high sensitivity, short response time, wider linear range, and good operational stability but has worse long-term stability. The developed ODAP-selective membrane sensor could be a useful alternative for ODAP determination in real samples. Therefore, our studies will continue using developed potentiometric ODAP-selective membrane sensor for detection of ODAP in* Lathyrus sativus *and other real samples.

## Figures and Tables

**Table 1:** The composition and potentiometric performance characteristics of the all solid-state contact ODAP-selective membrane sensor.

Parameter	ODAP-sensitive membrane sensor	
Membrane composition	%30.0 PVC	
	%65.0 o-NPOE	
	%2.5 ODAP-ionophore	
	%1.5 di-cyclo-hexyl 18-crown-6-NaI	
	%1.0 KTpClPB	
Detection limit (mol/L)	1.2 × 10^−6^	
Linear range (mol/L)	1.0 × 10^−1^–8.0 × 10^−6^	
Response time (s)	<6	
Linear equation	*y* = −34.6*x* + 2664.2	
Correlation coefficient (*r* ^2^)	0.9904	

	Interfering	(*k*_Odap,interfering_^pot^)

Selectivity coefficient (*k*_Odap,interfering_^pot^)	K^+^	1.88 × 10^−2^
	Na^+^	1.20 × 10^−2^
	Ca^2+^	3.96 × 10^−3^

*k*_Odap,interfering_^pot^: near interfering Na^+^, K^+^, and Ca^2+^ ions are reflected to exhibit selectivity to ODAP of sensor. The bigger the selectivity coefficient, *k*_Odap,interfering_^pot^, the smaller the sensitivity of the electrode towards ODAP measured.
